# Cholinergic Mechanisms in Gastrointestinal Neoplasia

**DOI:** 10.3390/ijms25105316

**Published:** 2024-05-13

**Authors:** Natalia Sampaio Moura, Alyssa Schledwitz, Madeline Alizadeh, Asha Kodan, Lea-Pearl Njei, Jean-Pierre Raufman

**Affiliations:** 1 Department of Medicine, Division of Gastroenterology and Hepatology, University of Maryland School of Medicine, Baltimore, MD 21201, USA; natalia.sampaiomoura@som.umaryland.edu (N.S.M.); alyssa.schledwitz@som.umaryland.edu (A.S.); akodan@som.umaryland.edu (A.K.); 2 The Institute for Genome Sciences, University of Maryland School of Medicine, Baltimore, MD 21201, USA; madeline.alizadeh@som.umaryland.edu; 3 Department of Biological Science, University of Maryland, Baltimore County, Baltimore, MD 21250, USA; lnjei1@umbc.edu; 4 Veterans Affairs Maryland Healthcare System, Baltimore, MD 21201, USA; 5 Marlene and Stewart Greenebaum Cancer Center, University of Maryland Medical Center, Baltimore, MD 21201, USA; 6 Department of Biochemistry and Molecular Biology, University of Maryland School of Medicine, Baltimore, MD 21201, USA

**Keywords:** acetylcholine, gastrointestinal cancer, cancer, brain–gut axis, muscarinic receptors, nicotinic receptors, cellular signaling, protein kinases

## Abstract

Acetylcholine-activated receptors are divided broadly into two major structurally distinct classes: ligand-gated ion channel nicotinic and G-protein-coupled muscarinic receptors. Each class encompasses several structurally related receptor subtypes with distinct patterns of tissue expression and post-receptor signal transduction mechanisms. The activation of both nicotinic and muscarinic cholinergic receptors has been associated with the induction and progression of gastrointestinal neoplasia. Herein, after briefly reviewing the classification of acetylcholine-activated receptors and the role that nicotinic and muscarinic cholinergic signaling plays in normal digestive function, we consider the mechanics of acetylcholine synthesis and release by neuronal and non-neuronal cells in the gastrointestinal microenvironment, and current methodology and challenges in measuring serum and tissue acetylcholine levels accurately. Then, we critically evaluate the evidence that constitutive and ligand-induced activation of acetylcholine-activated receptors plays a role in promoting gastrointestinal neoplasia. We focus primarily on adenocarcinomas of the stomach, pancreas, and colon, because these cancers are particularly common worldwide and, when diagnosed at an advanced stage, are associated with very high rates of morbidity and mortality. Throughout this comprehensive review, we concentrate on identifying novel ways to leverage these observations for prognostic and therapeutic purposes.

## 1. Introduction

Elegant pharmacological studies conducted by the esteemed British physiologist Sir Henry Dale exploring the differential actions of two naturally occurring alkaloids, nicotine and muscarine, revealed that acetylcholine (ACh)-induced cholinergic signaling can be divided into two major divisions: nicotinic and muscarinic receptor signaling [1]. These actions are mediated by structurally distinct ligand-gated ion channel nicotinic and G-protein-coupled muscarinic receptors, which can be further subdivided into multiple structurally related receptor subtypes. The use of tobacco products, the major source of nicotine for humans, has long been associated with an increased risk for a variety of cancers, especially those of the oropharynx and lung. Although these observations suggest a pro-neoplastic mechanism involving nicotinic cholinergic signaling, the role of nicotine per se as a carcinogen remains uncertain. Rather than initiating cancers, nicotine appears to function as a tumor promoter that stimulates the growth and expansion of cancer, whereas some of the many other components of tobacco products are likely to serve as cancer initiators [2].

Over the past 25 years, it has become evident that muscarinic cholinergic signaling promotes cancer progression [3]. Compared to nicotinic cholinergic signaling, the delay in recognizing the pro-neoplastic role of muscarinic signaling may have resulted from the observation that mushrooms, the major dietary source of muscarine, are not addictive and, consequently, do not share the popularity of tobacco use; unlike nicotine, muscarine does not cross the blood–brain barrier and has little effect on the central nervous system. Whereas the physiological activator of nicotinic and muscarinic receptors, ACh, is released rapidly at neural synapses and has a short duration of action due to efficient hydrolysis by acetylcholinesterases, neither nicotine nor muscarine are hydrolyzed by tissue or serum cholinesterases, thus prolonging their biological half-lives and resulting in effects that may not necessarily mimic those of ACh [4].

Over the past two decades, technological advances permitted the structures of many components of cholinergic signaling systems to be solved, thereby providing detailed information regarding key molecular interactions. The detailed structures of nicotinic and muscarinic ACh receptors (nAChRs and mAChRs, respectively) were obtained using cryoelectron microscopy [5]. Quantitative RT-PCR was used to investigate differential expression of various components of the cholinergic system in ACh-producing crypt-villus organoids lacking nerve and immune cells [6]. Notably, this technique is limited by its ability to measure RNA, rather than protein expression.

In this comprehensive review, we concentrate on the role of both nicotinic and muscarinic cholinergic signaling mechanisms in gastrointestinal (GI) neoplasia. After summarizing the mechanics of ACh synthesis and release, and the classification of ACh-activated receptors, we critically evaluate the evidence that constitutive and ligand-induced activation of these receptors plays a role in the induction and progression of GI neoplasia. We focus primarily on gastric, pancreatic, and colon adenocarcinomas, because these cancers are common and, if not resected surgically at an early stage, are associated with very high rates of morbidity and mortality. Throughout this review, we focus on opportunities to leverage this information for prognostic and therapeutic purposes.

## 2. Overview of Cholinergic Pathway

### 2.1. ACh Synthesis

**Figure 1:**
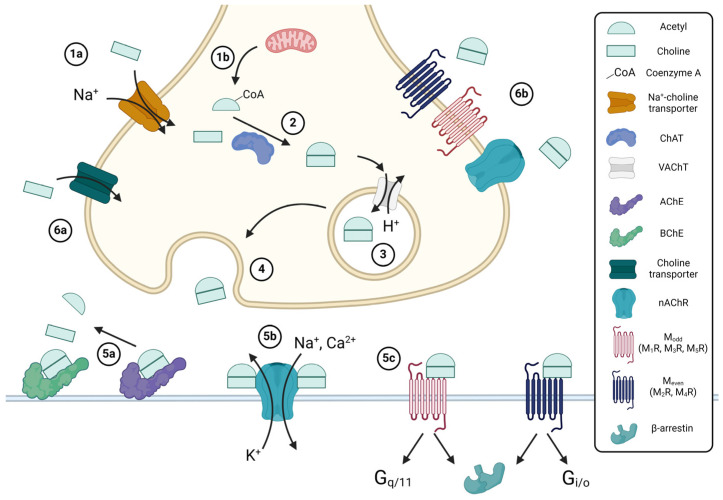
Overview of acetylcholine (ACh) synthesis. Choline enters cells via the sodium-coupled choline transporter (1a), whereas acetyl-coenzyme A (CoA) is produced in mitochondria from glycolysis or the metabolism of fatty acids (1b). Choline acetyltransferase (ChAT) catalyzes the synthesis of ACh from acetyl-CoA and choline (2); free CoA is a byproduct. ACh is loaded into vesicles by the vesicular ACh transporter (VAChT), a proton antiporter (3), and released by exocytosis (4) into either the synaptic cleft for neuronal ACh synthesis or the extracellular space for non-neuronal ACh synthesis. Prior to interacting with receptors, ACh may be hydrolyzed by acetylcholinesterase (AChE) or butyrylcholinesterase (BChE) (5a), forming acetate and choline; the resulting choline may then undergo reuptake (6a). ACh may bind to nicotinic (nAChR) (5b), muscarinic (mAChR) (5c), or other receptors, in a paracrine (5b/c) or autocrine (6b) fashion. The binding of two ACh molecules activates nAChR and opens a non-selective cation channel. Activated mAChRs can recruit either G proteins, which initiate either Gq/11 or Gi/o signaling, or G-protein-coupled receptor kinases, which recruit β-arrestins, leading to receptor desensitization via their internalization. Created with BioRender.com.

**Figure 2:**
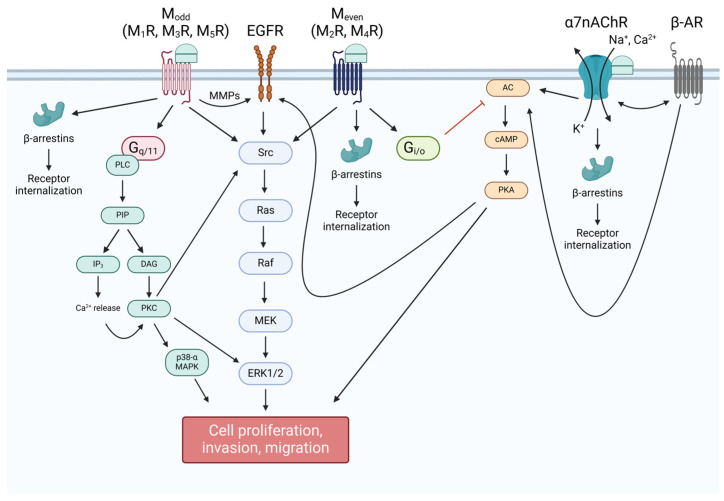
Overview of post-receptor acetylcholine (ACh) signaling. Odd-numbered muscarinic receptors (M_odd_: M_1_R, M_3_R, and M_5_R) are coupled to G_q/11_ signaling, which effects cellular change via phospholipid metabolism and increasing intracellular calcium concentrations, while even-numbered muscarinic receptors (M_even_: M_2_R and M_4_R) are coupled to G_i/o_, which inhibits the formation of cAMP by membrane-bound adenylyl cyclase (AC). As a result of M_even_ signaling, protein kinase C (PKC) is activated, which further activates mitogen-activated protein kinases (MAPK), such as p38 mitogen-activated protein kinase (MAPK) and extracellular signal-related kinase-1/2 (ERK1/2), to alter gene expression. ACh-bound M_odd_ also transactivates epidermal growth factor receptors (EGFR) via activation of matrix metalloproteinases (MMP), offering another mechanism whereby ERK1/2 can be activated. M_even_ activation is not known to directly transactivate EGFR, and instead leads to the inhibition of the activation of EGFR by protein kinase A (PKA). Nicotinic signaling is diverse and subtype-dependent. α7nAChR activates AC, leading to upregulation of cAMP production and PKA activation, both directly and through interplay with β-adrenergic receptors (β-AR). Muscarinic and nicotinic cholinergic receptors can also recruit β-arrestins, which mediate receptor internalization. Created with BioRender.com.

Produced in both central and peripheral nervous tissue, acetylcholine (ACh) acts as the primary neurotransmitter of the peripheral nervous system [7]. ACh acts a key excitatory neurotransmitter in the GI tract, predominantly modulating GI motility and secretions by stimulating both nicotinic and muscarinic ACh receptors [8]. Production of ACh is exclusively performed by choline acetyltransferase (ChAT), which catalyzes the joining of choline and acetyl-CoA in the axon terminals of neurons, where ChAT is predominantly localized (Figure 1) [9,10]. Choline, obtained from the diet, enters axon terminals via high-affinity Na^+^/choline transporters (CHT) [11]. Following its production, ACh is loaded into synaptic vesicles via vesicular ACh transporters (VAChT), which are proton exchange antiporters, before being released into the synapse by exocytosis. Notably, the gene encoding VAChT lies within the first intron of the gene encoding ChAT [7]. In contrast to most neurotransmitters, which undergo reuptake from the synapse in their secreted form, ACh is hydrolyzed by acetylcholinesterase (AChE) following release into the synaptic cleft [12], approximately half of the choline generated is taken up at nerve terminals by CHT, and the remainder diffuses extrasynaptically [13,14]. Intracellular cholinergic signaling pathways are diverse and vary by receptor, cell type, and environment, as exemplified in Figure 2.

ACh is produced and released from both neuronal and non-neuronal sources, including epithelial, endothelial, immune, and various cancer cells. Many other cell types express the components required to produce and release ACh without clear evidence of ACh production [15,16]. ACh is reportedly produced and released by several cell types in the epithelium of the GI tract, including tuft, immune, and crypt cells, as well as enteric neurons in the submucosal and myenteric plexuses, and resident immunocytes and gut microbiota cells [16,17,18,19,20,21,22]. ChAT splicing variants are produced by humans, but their tissue specificity is not well characterized [21]. Enteric ACh release is modulated by several factors, including epinephrine and the release of GI hormones [23]. Reduced esophageal ACh synthesis secondary to altered ChAT expression was observed in feline models of chronic idiopathic cystitis [24], indicating that disorders resulting from mutations altering ACh synthesis may have effects throughout the GI tract.

### 2.2. Nicotinic ACh Receptors

nAChRs are ionotropic transmembrane receptors expressed by both neuronal and non-neuronal tissue [25]. Both somatodendritic and presynaptic nAChRs located in myenteric neurons are responsible for cholinergic transmission, and play a role in mediating GI motility and secretion [26]. This occurs in tandem with mucosal nAChR modulation of these activities, whereby epithelial or other ACh production can activate nAChRs in various cell types (e.g., immune cells in the lamina propria) [27,28]. In the gut, ACh activation of nAChRs is associated with positive feedback loops that stimulate additional ACh release from myenteric motor neurons [26].

nAChR subtype heterogeneity results from combinations of 17 subunits, including α1–α10, β1–β4, δ, ε, and γ subunits, with corresponding gene designations (*CHRNA1-10*, *CHRNB1-4*, *CHRND*, *CHRNE*, *CHRNG*) [18,29]. Homomeric (α exclusively) and heteromeric nAChRs are all pentameric and differentially distributed in muscle and neuronal tissues. Whereas heteromers are found in both tissue types, homomeric receptors are exclusive to neuronal tissues; muscle heteromers are composed of α12β1γδ (embryonic) or α12β1εδ subunits, while neuronal nAChR heteromers are composed exclusively of α and β subunits [30]. α3, α4, α7, β2, and β4 are the most frequently observed subtypes in the GI tract, with α3 and β4 being the most common [31]. Along the GI tract, there is diverse nicotinic receptor subtype distribution, and varying sensitivity to ACh. For example, receptors containing β2 subunits are more sensitive to ACh than those containing the β4 subtype. In rats, the highest receptor levels are observed in the stomach and intestines, the majority of which are α3(α5)β4, α3β2, or homomeric α7 nAChRs [32], though α9α10 and α7α8 subtypes are also expressed [18].

GI nAChRs appear to have a complex relationship with mucosal inflammation. ACh stimulation of α7nAChR attenuates inflammatory cytokine production [33], which appears to come, in part, from inhibition of LPS-induced IL-1β stimulation [34] and NF-κB nuclear translocation, thus preventing downstream inflammatory cascade activation [35]. IL-1β stimulation also appears to inhibit ACh-driven contractility, suggesting a complex, dynamic relationship between IL-1β and ACh [36]. This could provide a potential therapeutic target for inflammation-associated dysplasia [37]. However, α_7_nAChR activation may also play an opposing role, and was shown to increase proliferation, migration, and metastasis in multiple digestive cancers [35]. Given their high concentration in the peripheral nervous system, and the ease with which compounds can be prevented from crossing the blood–brain barrier, nAChRs may prove a potent therapeutic target for digestive cancers. This is especially true if an appropriate partial agonist driving an anti-inflammatory and anti-neoplastic downstream cascade is identified.

### 2.3. Muscarinic ACh Receptors

mAChRs, within the large family of G-protein-coupled receptors (GPCRs), are integral components of cholinergic signaling in the GI system. Muscarinic receptors play pivotal roles in regulating a range of physiological processes, such as GI motility, secretion, and mucosal homeostasis, that are critical for the efficient functioning of the digestive system [38].

mAChRs are categorized into five structurally related subtypes, designated M_1_ through M_5_ (M_1_R–M_5_R), encoded by genes designated *CHRM1* through *CHRM5*. Each receptor subtype exhibits unique physiological roles and tissue-specific distribution. M_3_R, abundantly expressed by smooth muscle cells, neurons, and epithelial cells across the GI tract and pancreas, is instrumental in mediating cholinergic signaling, a crucial factor for maintaining normal GI functions [38]. Yet, some 25 years ago, M_3_R was also the first subtype identified as particularly important in the context of GI neoplasia [39].

The distribution of mAChRs within the GI tract exhibits remarkable variation in density and functionality across different regions. Notably, both M_2_R and M_3_R are highly concentrated in the muscle layers of the gut, playing a vital role in regulating peristalsis and other aspects of intestinal motility. Furthermore, these receptors contribute substantially to the enteric nervous system and, by modulating neurotransmission, modulate overall gut function. Within the GI mucosa, M_3_R expressed by epithelial cells is a critical controller of secretion and mucosal integrity, essential properties for intestinal barrier function that protect the organism against toxins and pathogens and foster homeostasis [38].

Recent advances in digestive disease research shed light on the involvement of mAChRs, particularly the M_3_R subtype, in the pathogenesis of GI neoplasia. M_3_R contributes to tumor development by multifaceted, complex mechanisms. Dysregulated mAChR signaling is implicated in a variety of key oncogenic processes, including cell proliferation, migration, and invasion. These features contribute substantially to the progression and aggressiveness of GI cancers. Aberrant expression of mAChRs was reported in several GI cancers, including gastric, pancreatic, and colorectal adenocarcinomas, highlighting their potential importance for the development and progression of neoplasia [38]. mAChRs can also profoundly affect the tumor microenvironment, influencing immune responses and angiogenesis. Understanding these pathways and their downstream effects is crucial for developing targeted therapies to disrupt mAChR-mediated tumorigenesis. Current research is intensely focused on elucidating these signaling pathways, with the aim of identifying novel therapeutic targets and strategies to combat GI cancers more effectively [38].

## 3. Critical Appraisal of Methods Used to Investigate Cholinergic Signaling

### 3.1. Technical Approaches to Measuring ACh Levels

Our understanding of cholinergic signaling has been refined by advances in methodology. It was previously difficult to measure tissue concentrations of ACh because it is quickly and efficiently hydrolyzed by serum and tissue cholinesterases [6,40]. Unlike other neurotransmitters, no currently available techniques can fix ACh to tissue for immunohistochemical quantification. Hence, different methods were needed to measure ACh levels and investigate its interaction with receptors.

ACh can be measured using chromatographic methods, such as high-performance liquid chromatography (HPLC), a frequently used method of quantifying ACh in biological samples, which separates ACh from other soluble components based on molecular size [41,42]. Mass spectrometry (MS) can be paired with LC to identify ACh based on the charge and mass of individual particles [43]. Alternatively, ACh can be sensed via fluorescence spectroscopy (FS), in which fluorescent probes are incorporated into the molecule. Surface-enhanced Raman spectroscopy (SERS), a more recently developed method, may detect single ACh molecules in a sample [44,45].

Microdialysis is commonly used to investigate ACh signaling by measuring the release of ACh into the extracellular fluid. Dialysates recovered after perfusion of living tissue can be analyzed using radioenzymatic, immunological, or spectroscopic assays [41]. Although microdialysis probes are larger than synaptic clefts, they can be used to measure extrasynaptic ACh. Investigators used this approach to estimate the quantity and speed of neurotransmitter release, degradation, and diffusion [46]. Amongst other limitations, microdialysis probe insertion induces tissue edema and hemorrhage; thus, caution is warranted, as this approach recovers ACh from inflamed and otherwise damaged tissue [47]. Further, microdialysis probes typically measure only neuronal ACh release, as administering tetrodotoxin (a voltage-gated sodium channel blocker) reduces extracellular ACh measured using microdialysis probes by more than 95%, even in the presence of a cholinesterase inhibitor [48].

Cell-based neurotransmitter fluorescent engineered reporters (CNiFERs) were used to detect ACh binding to muscarinic receptors. For this technique, HEK293 cells were engineered to express M_1_R fused with TN-XXL, a protein that upon binding to cytosolic calcium produces a yellow color detectable by fluorescence resonance energy transfer (FRET); control cells expressing TN-XXL alone produce a red color. Since native HEK293 cells express endogenous M_1_R, the investigators used a high-throughput fluorometric plate reader to screen for confounding receptor activation. To test ACh diffusion, investigators implanted CNiFERs into a rat frontal cortex and electrically stimulated the nucleus basalis magnocellularis (NBM). As NBM cholinergic fibers project into the neocortex, NBM stimulation shifted the spectral content recorded on the electrocortigram and CNiFERs responded in experimental, but not control, animals. Physostigmine, an acetylcholinesterase inhibitor, enhanced while atropine, a cholinergic antagonist, attenuated the CNiFER response [49]. These studies supported the use of genetically engineered cells to report cholinergic signaling but it is not evident that they can be applied to studies in the gut.

Other sensors were designed to monitor downstream effects of cholinergic signaling. One group used adeno-associated viral DNA to augment the genes for M_3_R with those for circular GFP (cGFP) to create a GPCR-activation-based ACh sensor (GRAB-ACh), which converts the ACh-induced conformational change in M_3_R into a fluorescent response. This model’s sensitivity was improved by focusing on the interface between the third intracellular loop of M_3_R and the cGFP residues, which contribute to its fluorescent intensity. Jing et al. compared the performances of the ACh2.0 and ACh3.0 sensors [50]. As control, they used a ligand-insensitive receptor (W200 mutation). ACh3.0 demonstrated an almost four-fold increase in fluorescence compared to ACh2.0, kinetic properties more like endogenous muscarinic receptors, and did not respond to stimulation with other neurotransmitters, e.g., nicotine. ACh3.0 sensors expressed in olfactory cells of transgenic Drosophila responded to odor stimulation, again with a substantially stronger two-photon imaging signal than ACh2.0. Similar findings were observed using two-photon imaging to track cholinergic signaling in the somatosensory cortex of mice given an object location discrimination task [50].

Another approach used ceramic-based microelectrodes coated with choline oxidase to detect changes in ACh concentration in a rat frontoparietal cortex. Measurements using these microelectrodes indicated that the rate of uptake for exogenous choline was reduced when the region was exposed to hemicholihium-3 (HC-3), a selective choline transporter (CHT) blocker. The microelectrodes revealed reduced choline clearance when CHTs were removed [51].

The above methods were used to address two competing theories regarding the nature of ACh transmission. The wired transmission model hypothesizes that ACh signaling occurs through direct synaptic communication, while the volume transmission model posits that ACh spreads diffusely [47]. Research findings support both theories, which are not mutually exclusive. Evidence supporting the wired transmission model demonstrates proximity between cholinergic neurons and mAChRs [52,53] and a rapid behavioral response during cue detection and auditory discrimination tasks [54]. Investigators using the ACh2.0 sensor in murine stellate neurons noted that fluorescent responses were restricted to clusters of individually isolated release sites, further supporting the wired transmission model [55]. Support for the volume model includes extrasynaptic M_1_R and M_2_R in the cortex with high affinity for ACh [56], and nAChRs dispersed diffusely along neural surfaces rather than clustered at discrete post-synaptic sites [57,58,59].

### 3.2. Current Challenges and Technical Advances in the Study of Cholinergic Signaling

In recent decades, advances in cholinergic signaling research uncovered several methodological and epistemological dilemmas. For bench research to have value, one must employ a physiologically accurate model system. GI cancer research introduces unique challenges due to the complexity of the tumor microenvironment and the many roles of ACh within that space. Once a system representation is established, the precision of data collection may be limited by available technology. Despite gains in our mechanistic understanding of cholinergic signaling, translation of that knowledge to clinical practice is hindered by obstacles in drug and biomarker development.

Replicating the GI cancer microenvironment makes it difficult to create a useful model to study cholinergic signaling. In some cases, a reductionist approach to system modeling is useful and appropriate. However, it is now known that a variety of cells and elements of the extracellular matrix contribute to cancer-related cholinergic signaling, including neurons, glial cells, gut microbiota, gut immunocytes, bile acids, surrounding epithelial and enteroendocrine cells, and metabolites [22]. In vitro, GI cancer cell co-culture with other cell types, such as neurons and glia of the enteric nervous system, allows for some consideration of cell–cell interactions, but still lacks the complexity of the tumor microenvironment.

Organoids are self-organized heterogenous tissue derived from patient tumors and/or stem cells, which model the complexity of an organ [60]. The use of patient-derived organoids (PDO) addresses some of the aforementioned concerns, as it allows for three-dimensional representation of the GI tumor microenvironment, including tumor–stroma interactions, and includes more soluble factors present in the tumor milieu. Others have reviewed the utility of PDOs in GI cancer research in greater depth [61]. With proper passaging, organoid systems can be cultured long-term, allowing for longitudinal studies of responses to various treatments. As personalized medicine advances, PDO models may prove useful in assessing individual drug sensitivities for targeted therapies [60]. Additionally, organoids are less prone to clonal evolution after multiple passages, a problem that plagues established cancer cell lines and contributes to data non-reproducibility [62,63,64]. If established cell lines are used, advances in telomere analysis now make it possible to serially assess the cell line for telomere attrition, a marker of clonal evolution [63,65]. While PDOs have the advantage of including multiple cell types, unless secondarily augmented, they lack the neural, immune, vascular, and microbial elements found in GI organs. There has been rapid innovation in developing system-on-a-chip technologies, essentially co-culturing organoids with other pre-selected cell lines, such as tumor-associated macrophages, fibroblasts, and gut microbiota, making it possible to model more complex tumor environments [66]. As reviewed elsewhere, this is being applied to research on the human gut microbiome and cancers of the stomach, liver, pancreas, and colon [67].

In vivo models present other limitations in the study of cholinergic signaling [22]. Patient-derived xenografts (PDX), wherein intact tumor cells from a human patient are implanted into a non-human animal, commonly mice, permit preclinical testing of potential treatments. This allows for the observation of the effects of the cancer and potential therapeutics on the whole organism, as opposed to observing only the cancer’s immediate environment, as in the organoid model. Compared to immortalized cancer cell lines or co-culture models, PDX models have the advantage of being less susceptible to genotypic drift. However, they are not immune to clonal evolution from the origin human tumor, likely due to differences in selection pressures in humans versus non-human model organisms [68,69,70]. Furthermore, compared to humans, non-human-derived models, particularly those using immunodeficient hosts, exhibit key differences in gut microbiota and innate immune components. Tumor engraftment rates in immunodeficient hosts are challengingly low, although advancements in the use of “humanized” mice, i.e., those transplanted with human hematopoietic stem cells and immune elements, may overcome this obstacle [70]. At present, given the short survival time of patients diagnosed with gastric, pancreatic, and late-stage colorectal adenocarcinomas, the time required to establish a PDX GI cancer model also limits its ability to benefit individual patients [71].

Regardless of the experimental model used, investigators face challenges related to the pharmacokinetics and pharmacodynamics of ACh and related reagents. As discussed in Section 3.1, several methods are available to quantify ACh and other molecules of interest in response to experimental treatments (Table 1). Challenges quantifying cholinergic signaling can be simplified into those related to spatial resolution, temporal resolution, and accuracy at low concentrations. Due to impediments to fixing tissue samples in a way that preserves free ACh, it is not possible to immunostain for ACh directly as it is for other small molecules. Instead, immunostaining for mAChRs or nAChRs can reveal the presence of those receptors in tissue with good spatial resolution. While this method allows for the visualization of AChR density over large areas of tissue, it cannot differentiate between ACh-bound and inactive receptors. Furthermore, staining specificity for cholinergic receptors is limited by antibody cross-reactivity and by the non-specific action of many cholinergic agonists and antagonists, which has made it difficult to study, for example, the divergent roles of M_1_R and M_3_R in colorectal carcinogenesis [72]. The concurrent use of multiple primary antibodies or proximity ligation assays increases detection specificity for M_3_R [72]. Other immunostaining strategies, such as those targeting ChAT, VAChT, or the choline transporter, may be appropriate if the goal is to describe the distribution of cholinergic components across a tissue that is thousands or tens of thousands of microns in length [73,74,75]. However, the expression of the machinery for ACh synthesis in a particular cell does not necessarily imply increased cholinergic signaling. In this context, the issue of temporal resolution refers to the ability of a test to illustrate changes in ACh signaling or its sequelae over time, for example, during exposure to an experimental treatment. Studying this is complicated by the constant hydrolysis of ACh by AChE, as well as by the Brownian motion of ACh in the extracellular space (Figure 1).

The accuracy of ACh quantification at low concentrations should be an important consideration in experimental design, as ACh concentrations in the peripheral nervous system, including the enteric nervous system of the GI tract, are in the range of 10–100 μM, and even lower in the serosal fluid of the GI tract; the use of supraphysiologic ACh concentrations can be misleading [19,76]. Microdialysis, as described in the previous section, can be used to detect physiological concentrations of ACh. After collecting the ACh-containing fluid of interest (which typically will also contain AChE), inhibitors of AChE are added to prevent ACh hydrolysis. While microdialysis can quantify ACh levels in living tissue while filtering out larger molecular weight molecules, excessive addition of AChE inhibitors can skew readouts to falsely high values [76]. High-performance liquid chromatography with electrochemical detection (HPLC-ED) is highly sensitive in detecting ACh from various types of sample preparations (e.g., tissue supernatants, serum, microdialysis samples), with a limit of detection of 5 nM, but it lacks both spatial and temporal resolution and is labor- and time-intensive [74,76].

## 4. Roles of Cholinergic Signaling in GI Neoplasia

### 4.1. General Roles in Neoplasia

Recent studies investigated the role of the cholinergic system in mediating cell proliferation and carcinogenesis. Research using Dclk-positive tuft cells demonstrates that cholinergic stimulation of the gastric epithelium induces nerve growth factor (NGF) expression, which promotes the development of neoplasia. Findings imply a positive feedback loop, with NGF production promoting even more cholinergic nerve growth [81]. Tuft cell ablation or inhibition of NGF signaling inhibit tumorigenesis through M_3_R and Yes-Associated Protein (YAP) suppression [82]. YAP, a co-factor in Wnt signaling, positively regulates cell proliferation, and must be activated for β-catenin-dependent cancer growth in various tissues via activation of stem cells [83]. The loss of Apc upregulates YAP, and it is suggested that Apc inactivation only fully induces Wnt signal targets if there is sufficient cholinergic signaling through M_3_R [82]. ChAT is expressed by tuft cells in mice and humans and is upregulated in carcinogenesis [84]. Dckl1-positive tuft cells in M_3_R-deficient mice compensate for the lost muscarinic signaling by secreting ACh [85]. Ablation of Dclk-positive cells inhibits epithelial proliferation [86].

There has been extensive research on the varying roles of different muscarinic receptor subtypes involved in GI neoplasia. It is generally thought that dysregulated MR signaling is associated with tumor progression, and neoplastic cells tend to interfere with MR-dependent proliferative signal transduction pathways [87,88]. Mouse models with sporadic and genetic colon cancers had reduced tumor burden when *Chrm3* was ablated, resulting in M_3_R deficiency [89]. However, because M_3_R deficiency reduces the number of adenocarcinomas rather than adenomas, it may be involved in neoplasia progression rather than initiation [82]. Conversely, M_1_R deficiency does not inhibit carcinogenesis. Indeed, mice with combined M_3_R and M_1_R deficiencies developed the same number of colon tumors as control mice, implying that M_1_R deficiency counteracts the effects of M_3_R deficiency [90]. Cholinergic interaction with nicotinic receptors has also been investigated. Studies using organoid systems indicate that ACh activates α2β4nAChR in Paneth cells, activating Wnt signaling and promoting stem cell proliferation and differentiation [91].

It is likely that other factors play a role in cholinergic receptor-induced promotion of cancer cell proliferation. Serotonin release from neurons in the enteric nervous system (ENS) promotes mucosal epithelial turnover by regulating muscarinic cholinergic innervation to epithelial cells; the mechanism of action remains unclear [86]. One study demonstrated that ACh inhibition with simultaneous serotonin upregulation returns crypt proliferation to its wild-type state, implying that serotonin effects on epithelial turnover require cholinergic modulation [86]. Due to structural similarities between ACh and bile acids, it is also suggested that molecular mimicry is involved in competition for receptor binding sites [92]. In pancreatic duct, stomach, and colon cells, it was found that normal and neoplastic epithelial cell function was altered via muscarinic mechanisms when cells were exposed to sustained elevated levels of bile acids [93]. The ENS has also been investigated regarding its role in stimulating cancer stem cell growth, promoting colon cancer invasion, and serving as a route for the physical dissemination of colon cancer cells [94].

Studies have explored the role of ACh in maintaining intestinal stem cell homeostasis, rather than only being active during cellular proliferation. Whereas one study showed that ACh signaling in intestinal organoids increased epithelial growth in an ENS-dependent manner [95], others demonstrated that the ablation of M_2_R, M_3_R, and M_5_R increased small intestinal epithelial cell proliferation, which suggests that ACh may inhibit epithelial cell turnover [96]. Other studies demonstrated that ACh treatment of intestinal organoids reduced cyclin D1 expression, a key factor mediating cell proliferation [97]. Ablation of the β4 nAChR subunit was shown to decrease crypt size and the number of intestinal stem and epithelial cells [96].

In the following sections, we will detail specific pathways activated by muscarinic signaling that allow for cellular proliferation, such as Wnt/Apc/β-catenin, MAPK/ERK, epidermal growth factor receptor (EGFR), and, for cellular migration, E-cadherin, matrix metalloproteinase (MMP), osteopontin, PI3K/AKT in gastric, pancreatic, and colon cancers.

### 4.2. Gastric Cancer

Gastric cancer is a major international health concern; in 2020 there were ~1.1 million cases and 770,000 deaths worldwide [98]. Gastric cancer initiation and progression can be influenced by modifiable risk factors, for example, cigarette smoking, excessive alcohol use, and most importantly *Helicobacter pylori* infection, which induces chronic gastritis and consequent intestinal metaplasia and dysplasia. Non-neuronal and neuronal cholinergic signaling contribute to gastric cancer development through activation of M_3_R and nAChRs. Yu et al. showed that ACh may be synthesized and released by gastric cancer cells, resulting in autocrine and paracrine activation of M_3_R, thereby inducing gastric cancer cell proliferation via transactivation of epidermal growth factor receptor (EGFR) signaling [99]. Compared to normal gastric epithelial cells, ChAT is overexpressed in human gastric cancer cell lines. Autocrine and paracrine ACh-induced activation of M_3_R and EGFR downstream signaling stimulates human gastric cancer cell proliferation [99].

As an important player in gastric tumorigenesis, M_3_R mediates the effects of ACh through different pathways and plays a role in cellular differentiation via Lgr5^+^ stem cells. *CHRM3* is overexpressed in human gastric cancer compared to paired normal tissue [100]. Murine studies suggest ACh signaling via this pathway in these stem cells is important for cancer initiation and progression [101]. Lgr5^+^ gastric stem cells are largely supported by ACh-producing nerves and tuft cells and expand in response to cholinergic signaling during carcinogenesis [102]. This occurs through an intricate sequence: increased enteric nerve expression in the stomach during tumorigenesis leads to augmented neuronal ACh production, which in turn leads to neoplastic upregulation of NGF and consequent clonal stem cell expansion. Further, post-M_3_R signaling may activate the Hippo/YAP axis, and consequently Wnt signaling, to further promote gastric cancer cell growth [82,103,104,105].

In turn, nAChRs may mediate the effects of cigarette smoking in gastric neoplasia. Two main tobacco components, nicotine and 4-(methylnitrosamino)-1-(3-pyridyl)-1-butanone (NNK), are selective nAChR agonists [106]. Though there is relatively less information on the impact of nAChR signaling compared to muscarinic signaling, two nAChR subtypes are well studied, α5 and α7. Compared to adjacent normal-appearing gastric tissue, α5nAChR expression is increased in gastric cancer, and activation of this receptor subtype induces human gastric cancer cell proliferation. Notably, nicotine curbs the chemotherapeutic effect of cisplatin by activating the Akt pathway through α5nAChR, promoting survival of cancer cells [107]. α7nAChR activation stimulates cell proliferation in an Erk1/2- and MAPK-dependent fashion and enhances gastric cancer cell migration by downregulating tumor suppressor gene E-cadherin and upregulating transcription factors ZEB-1 and Snail. These events promote tumor invasion and metastasis [106,108].

Several promising therapeutic avenues to ameliorate gastric cancer by leveraging the key role of muscarinic receptor signaling were proposed and tested in animal models. These include gastric cancer denervation by surgical vagotomy, M_3_R blockade, and inhibition of YAP signaling [109]. Murine studies revealed that vagotomy attenuates tumorigenesis in the denervated stomach and reduced cancer recurrence [102,109], which was very likely due to inhibition of Wnt and possibly EGFR signaling within gastric stem cells [99,103]. Specifically targeting M_3_R also attenuates gastric cancer. In vitro, M_3_R deficiency impeded gastric cancer cell proliferation via apoptosis [100]. In pre-clinical studies, selective M_3_R antagonism with 4-DAMP and darifenacin, FDA-approved for management of overactive bladder, markedly inhibited gastric tumor formation in vivo [99]. Small molecule inhibitors of YAP and its DNA-binding transcription factors, such as verteporfin and super-TDU, may also prove beneficial [110]. However, adverse effects, off-target toxicity, and therapeutic resistance may limit the benefits of these approaches; further research is required to explore their therapeutic potential for gastric cancer [111].

### 4.3. Pancreatic Cancer

Pancreatic ductal adenocarcinoma (PDAC), the tenth and ninth most common cancer in U.S. men and women, respectively, has an extremely low five-year survival rate: ~9% [112]. Due to PDAC’s aggressive course and nonspecific initial symptoms, more than half of cases have metastasized at diagnosis, at which point five-year survival is 3% despite aggressive immunotherapy and other new approaches. PDAC is markedly neurotropic; this is thought to favor progression [113,114].

Nicotinic signaling is especially germane to PDAC development and progression. It is well established that nicotinic ACh receptor agonists promote PDAC; since the first studies associated tobacco use with pancreatic cancer in 1973, an abundant literature describes mechanistic links focusing on nicotine and nicotine-derived nitrosamine ketone (NNK) [115,116]. Exposure to cigarette smoke activates stem cell features of both benign and malignant pancreatic cells via α7-nAChR signaling and causes pancreatic acinar cell dedifferentiation via loss of the GATA6 protein [117,118]. Furthermore, signaling through nAChR subtypes α3, α4, α5, and α7 stimulates PDAC stem cells to proliferate and resist tumor suppression by GABA [119]. Selective knockdown of α4β2nAChR inhibits GABA production by PDAC cell lines and benign pancreatic epithelial cells [120]. Nicotinic signaling also induces cellular dysplasia via oxidative stress and hypermethylation of tumor suppressor genes, including *PENK*, which encodes proenkephalin [121,122]. When early PDAC is established, α7nAChR activation promotes cell survival and proliferation through MAPK and Ras-Raf-MEK-ERK signaling [121,123]. In a large study of murine pancreatic cancer induced by 7,12-dimethylbenzanthracene (DMBA), subcutaneous nicotine administration greatly enhanced PDAC incidence [124]. Interestingly, there was no difference in the incidence of pancreatic intraepithelial neoplasia (PanIN), the precursor to PDAC, between the two groups, suggesting that nAChR activation is consequential in the progression of PanIN to the highly aggressive PDAC. This is supported by the finding that exposing PDAC cells, whether a human cell line or an orthotopic xenograft model, to nicotine doses comparable to those experienced by tobacco smokers stimulates tumor proliferation by downstream activation of α3, α5, and α7nAChRs, which results in the production of epinephrine and norepinephrine [125,126]. RNA interference knockdown of α3, α5, or α7nAChRs, but not that of α4nAChRs, results in decreased catecholamine synthesis in response to a nicotine treatment in both benign pancreatic epithelial cells and PDAC cells, highlighting their roles in PDAC development via beta-adrenergic crosstalk [125]. Longitudinal treatment of PDAC and pancreatic epithelial cell lines with nicotine results in sensitization of the α3, α4, α5, and α7nAChR-beta-adrenergic signaling connection, and increased cell migration [120]. Others have described the noteworthy interplay between beta-adrenergic signaling and PDAC progression [127].

As PDAC progresses, activation of α7nAChR induces expression of osteopontin, a cytokine and component of the ECM, by PDAC cells, which in turn causes PDAC cells to take on a more migratory and invasive phenotype [128]. Proposed mechanisms for this include activation of the PI3K/AKT/NFκB axis and activation of matrix metalloproteinase (MMP)-2. Others have found that nicotinic signaling augments PDAC invasiveness in a Src-dependent manner by upregulating MUC4 mucin expression [129,130]. α7nAChR agonism also contributes to PDAC metastasis through activation of JAK2/STAT3 and MEK-ERK signaling [129]. Interestingly, the endogenous α7nAChR ligand Secreted Ly-6/uPAR-Related Protein 1 (SLURP1) appears to oppose the carcinogenic downstream effects of ACh or other nicotinic agonists binding to α7nAChR in PDAC. The anti-proliferative features of SLURP1 may stem from its inhibition of multiple oncogenes and pro-inflammatory cytokines [131]. As for other cancers, the ubiquity of nicotinic and muscarinic ACh receptors in bodily tissues, and the resulting risk of off-target effects, has hindered cholinergic-targeted therapy development for pancreatic cancer [116].

Nicotinic signaling contributes in other meaningful ways to pancreatic cancer. Chronic nicotine exposure causes PDAC cell resistance to the first-line chemotherapeutic agent gemcitabine [132]. In PDX models of PDAC, further exposure to nicotine also promoted cancer-induced cachexia in an IL-8- and ERK-dependent fashion [133].

Relatively less is known about the role of muscarinic signaling in PDAC. In a single-center study examining 58 human PDAC samples, M_3_R was overexpressed in every sample compared to patient-matched normal pancreatic tissue, with the intensity of M_3_R staining positively correlating with tumor grade and the presence of lymph node metastases, although interestingly not with distant metastasis, and negatively correlating with overall survival [134]. In a secondary analysis, the highest levels of M_3_R expression were at the invasive tumor front and lymph node metastases, suggesting a role for M_3_R in active tumor invasion rather than in more differentiated PDAC cells.

M_1_R appears to counter PDAC progression, although the relevant downstream targets of M_1_R activation are unknown. In a mouse model, treatment with the nonselective muscarinic agonist bethanechol reduced PDAC stemness and progression, and increased longevity [113]. These effects appeared to be M_1_R-activation-dependent. A non-selective muscarinic receptor agonist, bethanechol is currently in phase 1 clinical trials as a pre-operative treatment for PDAC [135]. Identifying PDAC genes altered by muscarinic signaling is an active area of investigation.

### 4.4. Colorectal Cancer

In the U.S., colorectal cancer (CRC) remains the second most common cause of cancer death. Moreover, recent years have witnessed a substantial increase in the proportion of individuals younger than 55 years old diagnosed with CRC [136,137]. As in gastric and pancreatic cancer, M_3_R is overexpressed in human colon cancer cells compared to healthy colonocytes. Different studies interrogated M_3_R expression on primary colon cancer cells and primary colon cancer tissue, utilizing techniques such as reverse transcription-PCR and tissue immunostaining. Yang et al. reported up to eight-fold increased M_3_R expression in primary colon cancer cell lines [138], and Cheng et al. reported up to two-fold increased tissue M_3_R staining intensity in cancer compared to adjacent normal colon [139]. M_3_R overexpression is associated with increased cell proliferation and CRC progression [139,140], findings further supported by murine studies showing that global M_3_R deficiency results in fewer colon tumors compared to littermate controls [90]. Of the other four muscarinic subtypes, M_1_R expression is also altered in human colon cancer. Analysis of publicly available RNA-seq data from CRC tissue samples and paired normal tissue revealed reduced *CHRM1* expression in CRC [140]. Likewise, murine studies suggest that M_1_R expression may suppress the pro-neoplastic impact of M_3_R activation [90].

Two physiological ligands are implicated in M_3_R activation in CRC, ACh, and bile acids. Quantitative real-time PCR and immunostaining provided evidence of ChAT expression in both human colon cancer cells and colon cancer tissue specimens, and treatment of human colon cancer cells with acetylcholinesterase inhibitors increased their proliferation in vitro [74]. These findings suggest that in addition to other neuronal and non-neuronal sources of ACh, colon cancer cells can produce and release ACh, thereby adding autocrine and paracrine stimulation of cell proliferation through M_3_R to the cancer cell repertoire [74,141]. Epidemiological and murine studies provided evidence that fecal bile acids, particularly lithocholic and deoxycholic acids, are associated with the development and progression of CRC [142,143,144]. Targeted studies revealed that selected bile acids stimulate proliferation of colon cancer cells via activation and transactivation, respectively, of M_3_R and EGFR [145,146].

**Figure 3:**
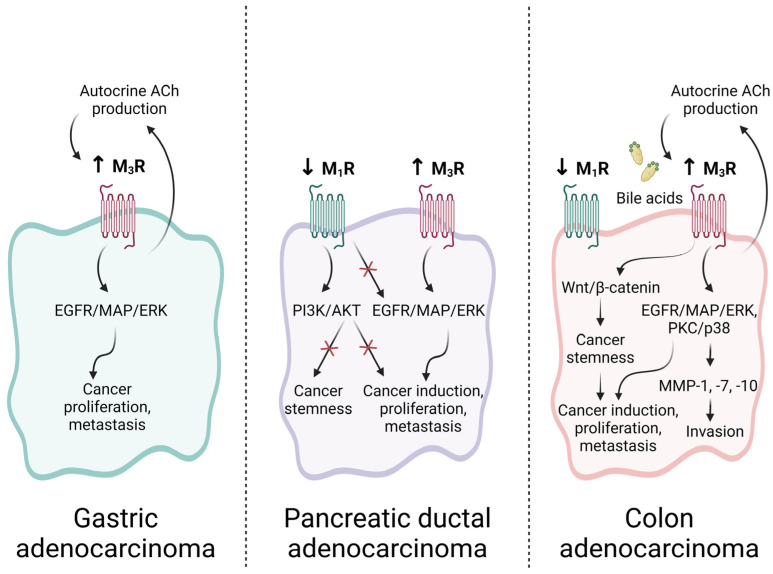
Roles of muscarinic signaling in GI cancer. Gastric and colon cancer cells produce non-neuronal ACh, which is implicated in autocrine and paracrine signaling. In gastric adenocarcinoma, M_3_R is overexpressed relative to normal gastric epithelial cells, and M_3_R activation stimulates gastric cancer progression and metastasis via an EGFR/MAP/ERK-dependent process. In pancreatic ductal adenocarcinoma (PDAC), M_3_R is likewise overexpressed and associated with both cancer initiation and progression. Conversely, M_1_R expression is downregulated in PDAC, and opposes the carcinogenic roles of M_3_R both directly via inhibition of EGFR transactivation, and indirectly via inhibition of the cancer stem cell compartment. In colon cancer, activation of M_3_R, either by ACh or bile acids, promotes expansion of the cancer stem cell compartment via Wnt signaling and promotes tumor invasion and growth by upregulating matrix metalloproteinase (MMP) expression. M_1_R expression and activity are downregulated in colon cancer and M_1_R signaling is associated with reduced colon cancer cell proliferation by currently unknown mechanisms. Created with BioRender.com.

Common pathways mediate the actions of ACh and bile acids on CRC progression. Using different human colon cancer cell lines that express M_3_R, Cheng et al. and Ukegawa et al. reported that the interactions of both ACh and secondary bile acids with M_3_R resulted in transactivation of EGFR and downstream phosphorylation of key mediators, including ERK1/2, Akt, and p38 MAPK [146,147,148], provoking cell proliferation and survival. Through its effects in the EGFR pathway, increased cholinergic signaling also upregulates the immunosuppressive molecule PD-L1, which impairs host immune surveillance. Kuol et al. reported that cholinergic blockade with atropine downregulated PD-L1 in cancer cell lines, highlighting potential crosstalk between immunosuppressive molecules and cholinergic signaling [141]. Tumor cell invasion may be promoted by M_3_R activation via induction of MMPs [149]. An overview of involved pathways in colon, gastric, and pancreatic cancers is shown in Figure 3.

Alternative muscarinic agonist-activated pathways can promote CRC. These include the finding that muscarinic agonists modulate β-catenin signaling and induce cyclooxygenase-2 (COX2) and consequent prostaglandin E-2 (PGE2) formation [138,150]. These are known GI cancer promoters [151]. Additionally, in human colon cancer cell lines, ACh-induced M_3_R activation selectively upregulates oncogenic microRNAs (miR-21, miR-221, and miR-222) [152].

In addition to muscarinic receptors, nAChR activation can promote CRC. α7nAChR is a major subtype of nAChRs, regulating proliferative and anti-apoptotic properties related to tumorigenesis [35,153]. α5nAChR plays a relatively smaller role in CRC tumorigenesis compared to α7nAChR, while other nAChR subunits implicated in the progression of other cancers, such as α5, β2, and β4 in lung cancer, do not seem to be involved in CRC [154]. ACh is the predominant endogenous ligand for α7nAChR and an autocrine and paracrine growth factor [155]. Several groups used immunohistochemistry, RT-PCR, and immunoblotting to reveal α7nAChR expression in HT-29 human colon cancer cells [155,156]. Nicotine-containing tobacco use is an established CRC risk factor [157]. Activation of α7nAchRs by nicotine contributes to CRC cell proliferation via MAPK/ERK signaling [156,158], inhibition of apoptosis [159], stimulation of CRC cell migration by fibronectin, MMP-1, -2, and -9 induction [158,160], and angiogenesis [157]. Similarly, nicotine-driven activation of α5nAchRs leads to increased cancer cell proliferation and migration through epithelial-to-mesenchymal transition [161]. Paradoxically, Fei at al. reported an anti-inflammatory role for α7nAchR expressed in CRC-associated macrophages, wherein downregulation of JAK2/STAT3 signaling may attenuate tumor metastasis [162]. A deeper understanding of α7nAchR expression and activation in colon cancer and the tumor microenvironment will provide additional insights.

Mechanistic insights into the role of muscarinic and nicotinic ACh receptor signaling in colorectal tumorigenesis can inform the development of novel anti-cancer modalities. M_3_R blockade in human colon cancer cell lines counteracted ACh-induced ERK1/2, Akt, and MMP-1 signaling, consequently attenuating cell proliferation and invasion [149]. Further, M_1_R expression is downregulated in human CRC tissue and may protect colon cells against neoplastic transformation [163]. In human colon cancer cell lines, Sundel et al. demonstrated that M_1_R agonism inhibits cell proliferation and augments the effects of conventional chemotherapeutic agents [163]. Likewise, α7nAchR antagonism reverses the stimulatory actions of nicotine on colon cancer cell proliferation [156].

## 5. Leveraging Cholinergic Signaling to Treat GI Neoplasia

### 5.1. Subtype-Selective Muscarinic Receptor Agonists or Antagonists

Given the contributory role of cholinergic signaling in GI neoplasia, the potential benefits of targeting muscarinic receptors to curb cancer progression is evident. Nonetheless, there are numerous obstacles to achieving this goal safely and effectively. A relative paucity of selective muscarinic receptor agonists and inhibitors results from the high degree of homology between subtypes. The development of global and conditional muscarinic receptor knockout mouse models and advances in altering gene expression permitted the development of receptor subtype-specific ligands [164]. Drugs targeting M_1_R and M_3_R were shown to impact GI cancer progression (Table 2). Whereas non-subtype-selective muscarinic agonists such as carbamylcholine (carbachol) and bethanechol stimulate cancer cell proliferation [138,141,148,163], M_3_R antagonists attenuate cancer proliferation in vitro and in vivo.

Specific M_3_R antagonists studied in the context of GI neoplasia are darifenacin hydrobromide, 1,1-dimethyl-4-diphenylacetoxypiperidinium iodide (4-DAMP), and aclidinium bromide. In 2004, darifenacin (brand name Enablex^®^) was approved by the U.S. Food and Drug Administration (FDA) to manage overactive bladder in women. Following oral administration, darifenacin is rapidly and nearly completely absorbed with few adverse effects [165]. In murine preclinical studies, darifenacin and 4-DAMP inhibit gastric tumor formation [99], reduce primary CRC volume, invasion, and weight [149], and induce prominent inhibition of colon cancer cell proliferation and viability [166]. In 2012, an inhaled form of aclidinium bromide (brand name Tudorza^®^), a long-acting M_3_R antagonist, was FDA-approved for chronic obstructive pulmonary disease [167]. Using gastric cancer cells, Wang et al. discovered that increasing concentrations of aclidinium bromide inhibited cell proliferation and pro-proliferative phosphoinositol 3-kinase signaling, and induced apoptosis [168].

The potential of targeting M_1_R expression in GI neoplasia and general limitations of muscarinic receptor therapy should also be considered. Since mouse models of M_1_R deficiency accelerated pancreatic tumorigenesis [169] and increased CRC burden [90], M_1_R agonism appears to be a promising approach for GI neoplasia. McN-A-343 and xanomeline, selective M_1_R agonists studied in the context of GI neoplasia, were developed decades ago, McN-A-343 in 1961 [170] and xanomeline in 1997 [171]. In vitro, McN-A-343 and xanomeline dose-dependently inhibited colon cancer cell proliferation, an effect reversed by pre-treating cells with a selective M_1_R inhibitor [163]. Moreover, adding McN-A-343 to standard colon cancer chemotherapy, namely 5-fluorouracil and oxaliplatin, potentiated their anti-proliferative effects on colon cancer cells [163].

Potential limitations to the use of therapies targeting muscarinic receptors include off-target effects and dose-dependent toxicity. M_1_R and M_3_R are co-expressed in both normal and neoplastic intestinal epithelial cells, and therefore unwanted anti-cholinergic side effects are anticipated. Moreover, clinically effective doses of M_1_R agonists and M_3_R antagonists for GI cancer therapy are currently unknown [172]. Nevertheless, given the work that demonstrates the successful use of M_3_R antagonism and M_1_R agonism in attenuating GI cancer progression in vitro and in vivo in preclinical animal models, their use as adjuvants to current anti-cancer therapies warrants exploration.

### 5.2. Nicotinic Receptor Antagonists

The potential for using nicotinic receptor antagonists to treat GI neoplasia is unclear. Selective α7nAChR antagonism using α-bungarotoxin, derived from snake venom [173], and methyllycaconitine (MLA), a naturally occurring alkaloid [174], attenuates GI cancer growth. Both α-bungarotoxin and MLA inhibited endothelial cell proliferation and angiogenesis [153]. MLA attenuated nicotine-induced human colon cancer cell proliferation [156]. Moreover, in two different gastric carcinoma cell lines, by blocking α7nAChR activation, rabies virus glycoprotein, produced by the non-virulent Newcastle disease virus, attenuated cell proliferation and migration [175,176]. Nonetheless, disruption of α7nAChRs may alter intestinal homeostasis by blocking its anti-inflammatory role; ACh-activated α7nAChRs expressed on immune cells suppress pro-inflammatory cytokine production [35]. Even though α7nAChR agonism with AR-R17779 and GSK1345038A was not protective in a murine colitis model [177], increased central ACh via treatment with a cholinesterase inhibitor attenuated colitis and reduced the risk of colitis-associated cancer due to activation of the cholinergic anti-inflammatory pathway via α7nAchR [178]. Thus, α7nAChR antagonism in immune cells could compromise the cholinergic anti-inflammatory pathway with detrimental effects. Thus, as with other therapeutic strategies directed at ACh-activated receptors, the key remains receptor selectivity, avoiding off-target adverse effects. Modulating α7nAChR activity may be promising if specific targeting of α7nAChRs on cancer cells can be achieved.

**Table 2:** Drugs targeting cholinergic signaling in GI neoplasia.

Receptor Selectivity	Compound	Brand Name	Therapeutic Application	GI Cancer Use	References
M_3_ antagonist	Darifenacin hydrobromide	Enablex	Overactive bladder	Inhibition of gastric and colorectal tumor formation and invasion	[99,149,165,166]
	Aclidinium bromide	Tudorza	Chronic obstructive pulmonary disease	Inhibition of gastric cancer cell proliferation	[167,168]
M_3_ > M_1_ > M_2_ antagonist	1,1-dimethyl-4-diphenylacetoxypiperidinium iodide (4-DAMP)	NA	Experimental inhibitor	Inhibition of gastric and colorectal tumor formation and invasion	[99,166,179]
M_1_, M_4_ agonist	McN-A-343	NA	Experimental agonist	Inhibition of colon cancer cell proliferation	[163,170]
	Xanomeline	KarXT (in combination with Trospium)	Alzheimer’s disease, Schizophrenia (FDA approval pending)	Inhibition of colon cancer cell proliferation	[163,171,180]
α7nAChR antagonist	Methyllycaconitine	NA	Experimental inhibitor	Inhibition of endothelial cell proliferation, blood vessel formation, and nicotine-stimulated colon cancer cells	[153,156]
Non-subtype-selective nAChR antagonist	Rabies virus glycoprotein	NA	Experimental inhibitor	Inhibition of gastric cancer cell proliferation and tumor migration	[175,176]

NA, not applicable.

## 6. Conclusions and Future Directions

As reviewed herein, numerous lines of investigation support the concept that nicotinic and muscarinic cholinergic receptor activation promotes GI cancer progression, primarily those of the stomach, pancreas, and colon. This is noteworthy because cancers originating from these three organ systems are increasingly common at a younger age and usually lethal if not diagnosed and excised at an early stage. Nonetheless, despite greater clarity regarding the underlying mechanisms of ACh production and release, and the signaling pathways downstream of muscarinic cholinergic receptor activation, key technical limitations remain problematic. Chief amongst these are limitations in the methodology available to measure serum and tissue ACh levels accurately in real time. Hence, we devoted considerable space to review methods for measuring ACh levels. All are limited by complexity and accessibility, thereby restricting their use; moreover, many of these approaches perturb experimental systems, thereby providing results that fail to measure actual ACh concentrations in health or disease with acceptable accuracy.

Additionally, leveraging the findings outlined in this review for prophylactic or therapeutic purposes remains a major challenge. In addition to approaches to impeding the activation of cholinergic receptors and of downstream molecules that mediate signal transduction and changes in cell function, prospective targets for therapeutic intervention include the cellular machinery required for ACh synthesis and release by neuronal and non-neuronal cells in the tumor microenvironment. To us, it appears that a crucial conundrum is how to selectively target cholinergic receptors and/or post-receptor signaling molecules solely in neoplastic cells and the tumor microenvironment without impacting normal cell function in the GI tract and other organ systems. The ubiquity of cholinergic receptor expressions and their structural similarity facilitates off-target effects that limit their use. The development of muscarinic receptor subtype-selective ligands, both activators and inhibitors, has been a major advance in the field, but targeting tumors specifically remains a daunting challenge.

Another challenge is identifying the stages of GI neoplasia most amenable to therapeutic interventions targeting cholinergic signaling. For example, adenomas or adenocarcinomas of the colon are commonly excised endoscopically or at surgery. Therefore, chemo- and immuno-therapeutics are generally reserved for GI cancer metastases, most commonly to the liver. Nonetheless, an exception may be persons with heritable forms of GI neoplasia, e.g., familial adenomatous polyposis (FAP). In that setting, treating FAP patients with agents that inhibit the production of COX2, a key promoter of colon neoplasia, may be a useful analogy. These agents proved effective in this small subset of persons with hereditary colon neoplasia [181,182,183]. Notably, M_3_R overexpression in aberrant crypt foci in colon tissues from FAP patients suggests that M_3_R activation promotes colon neoplasia in this disorder [139]. Thus, studies that test the efficacy of selective M_3_R antagonists, alone or in concert with COX2 inhibitors, to diminish both the formation of adenomas and their progression to advanced neoplasia in this population may be fruitful. Likewise, based on the information provided in this review, phase 2 or 3 studies to test the safety and efficacy of FDA-approved selective M_3_R antagonists, e.g., darifenacin, either alone or in combination with the standard-of-care, in persons with metastatic gastric, pancreatic, and colon cancers may be warranted. Similar studies could be considered for selective M_1_R agonists [140].

Finally, as evidenced by the long list of references accompanying this review, many research groups have contributed to this important area of investigation. Yet, despite the comprehensive nature of this review, due to space limitations and inadvertent oversights, we undoubtedly failed to review or cite impactful work by investigators in the field—we apologize for any such omissions.

## Figures and Tables

**Table 1:** Comparison of methodologies used to study muscarinic cholinergic signaling.

Method	Description	Pros	Cons	Refs
Chromatographic and spectroscopic methods, e.g., HPLC, LC-MS, FS, SERS	ACh-containing liquid sample is allowed to flow through stationary please column, where various components of the sample separate based on properties, e.g., molecular size and charge. The eluant can then be analyzed by spectroscopy, e.g., MS, SERS.	Relatively high accuracy and speed of processing. Some (e.g., LC-MS, SERS) have high sensitivity at low [ACh]. Does not destroy the sample in process of analysis. LC-MS can simultaneously identify other components of interest in addition to ACh in sample.	Spatial and temporal resolutions are poor, unless repeatedly analyzing ACh samples taken from various tissue locations and at different time points. False positives may occur due to detection of a carnitine precursor that shares the molecular formula of ACh.	[41]
Microdialysis	Probe and semi-permeable membrane inserted in tissue of interest. Fluid pumped through the probe and ACh diffuses across membrane while limiting movement of other molecules. Dialysate is analyzed to measure [ACh] via high-performance liquid chromatography (HPLC) or capillary electrophoresis (CE).	Calculates synaptic ACh using its recovery from extracellular space and allows in vivo measurement of ACh release with high spatial resolution.	Probe-related tissue injury impacts ACh release. Does not account for non-neuronal ACh. High detection limits require high [ACh] to register readings. Synaptic ACh calculated, not directly measured. Spatial resolution limited by probe size. Limited temporal resolution.	[47,48,76]
Secondary-messenger-based sensors (CNiFERs)	Cell-based neurotransmitter fluorescent engineered reporters (CNiFERs) detect downstream activity of ACh. Upon binding to cytosolic calcium, fusion protein expresses fluorescent color detected by fluorescence resonance energy transfer (FRET).	Allows in vivo measurement of ACh release, albeit invasively. Both neuronal and non-neuronal ACh are detectable. High temporal resolution.	Limited spatial resolution. Must screen cells for confounding receptor activity. Sensitivity limited by ACh receptor affinity and receptor desensitization.	[49,77,78]
GPCR-activation-based sensors (ACh2.0, ACh3.0)	Engineered viral DNA incorporated into host animal genome, allowing for conversion of ACh-induced M_3_R conformational changes into a fluorescent response. More recent modifications are focused on interface of the third intracellular loop of M_3_R and cGFP residues.	Sensitivity improved in newer model. In vivo non-invasive, high temporal resolution. Can be combined with high resolution imaging to gather additional neurotransmitter properties like the number of release sites.	Limited to M_3_R analysis with limited spatial resolution. Must screen cells for confounding receptor activity. Current designs only rapid enough to detect slow neurotransmitter release due to low/moderate physiological stimulation. Sensor performance can be affected by agonists or antagonists that bind to endogenous GPCRs.	[50,55,79,80]

HPLC, high-performance liquid chromatography; LC-MS, liquid chromatography with mass spectrometry; FS, fluorescence spectroscopy; SERS, surface-enhanced Raman spectroscopy; CNiFER, cell-based neurotransmitter fluorescent engineered reporters; GPCR, G-protein-coupled receptors; ACh, acetylcholine; Refs, references.
